# Persistence of amygdala hyperactivity to subliminal negative emotion processing in the long-term course of depression

**DOI:** 10.1038/s41380-024-02429-4

**Published:** 2024-01-26

**Authors:** Melissa Klug, Verena Enneking, Tiana Borgers, Charlotte M. Jacobs, Katharina Dohm, Anna Kraus, Dominik Grotegerd, Nils Opel, Jonathan Repple, Thomas Suslow, Susanne Meinert, Hannah Lemke, Elisabeth J. Leehr, Jochen Bauer, Udo Dannlowski, Ronny Redlich

**Affiliations:** 1 Institute for Translational Psychiatry, University of Münster, Münster, Germany; 2 Department of Psychiatry and Psychotherapy, Jena University Hospital, Jena, Germany; 3 German Center for Mental Health (DZPG), Halle-Jena-Magdeburg, Germany; 4 Center for Intervention and Research on adaptive and maladaptive brain Circuits underlying mental health (C-I-R-C), Halle-Jena-Magdeburg, Germany; 5 Goethe University Frankfurt, University Hospital, Department of Psychiatry, Psychosomatic Medicine and Psychotherapy, Frankfurt, Germany; 6 Department of Psychosomatic Medicine and Psychotherapy, University of Leipzig Medical Center, Leipzig, Germany; 7 Institute for Translational Neuroscience, University of Münster, Münster, Germany; 8 Department of Clinical Radiology, University of Münster, Münster, Germany; 9 Department of Psychology, Martin-Luther University of Halle, Halle, Germany

**Keywords:** Depression, Neuroscience

## Abstract

Biased emotion processing has been suggested to underlie the etiology and maintenance of depression. Neuroimaging studies have shown mood-congruent alterations in amygdala activity in patients with acute depression, even during early, automatic stages of emotion processing. However, due to a lack of prospective studies over periods longer than 8 weeks, it is unclear whether these neurofunctional abnormalities represent a persistent correlate of depression even in remission. In this prospective case-control study, we aimed to examine brain functional correlates of automatic emotion processing in the long-term course of depression. In a naturalistic design, *n* = 57 patients with acute major depressive disorder (MDD) and *n* = 37 healthy controls (HC) were assessed with functional magnetic resonance imaging (fMRI) at baseline and after 2 years. Patients were divided into two subgroups according to their course of illness during the study period (*n* = 37 relapse, *n* = 20 no-relapse). During fMRI, participants underwent an affective priming task that assessed emotion processing of subliminally presented sad and happy compared to neutral face stimuli. A group × time × condition (3 × 2 × 2) ANOVA was performed for the amygdala as region-of-interest (ROI). At baseline, there was a significant group × condition interaction, resulting from amygdala hyperactivity to sad primes in patients with MDD compared to HC, whereas no difference between groups emerged for happy primes. In both patient subgroups, amygdala hyperactivity to sad primes persisted after 2 years, regardless of relapse or remission at follow-up. The results suggest that amygdala hyperactivity during automatic processing of negative stimuli persists during remission and represents a trait rather than a state marker of depression. Enduring neurofunctional abnormalities may reflect a consequence of or a vulnerability to depression.

## Introduction

Studying the course of depression and its etiologic and maintaining factors has the potential to reduce its high burden of disease [1]. Cognitive models of depression suggest that onset and maintenance of depression are associated with biases in emotion processing operating throughout all stages of attention, memory, and evaluation, favoring the processing of negative emotional stimuli (cognitive vulnerability; [2]). Furthermore, according to Teasdale’s differential activation hypothesis, depressed mood in turn activates negative thought patterns (cognitive reactivity), leading to a negative reciprocal build-up process that creates a vulnerability to persistent depression and depressive relapse [3].

Accordingly, behavioral studies demonstrated a negative bias in attention, interpretation, and memory in depressed patients [4–6] as well as in individuals at risk of developing depression [7, 8]. These cognitive biases were found to operate even in early, automatic stages of processing, as demonstrated using subliminally presented stimuli [9]. In addition, a negative bias in emotion processing has been associated with future depressive relapse [10–12]. However, most of the behavioral studies have examined the controlled, later stages of emotion processing, which may be influenced by cognitive control and emotion regulation processes.

Recently, cross-sectional neuroimaging studies provided evidence that emotion processing in depressed patients is biased already in the early, automatic stages, pointing to brain functional alterations mainly in the amygdala, a key region of the emotion processing and salience network [13, 14]: Consistently, depressed patients show amygdala hyperactivity to masked, subliminally presented negative stimuli and a hypoactivity to positive stimuli compared to healthy subjects [15–17]. Furthermore, higher automatic amygdala reactivity to negative primes has been associated with a negative judgmental bias to the neutral target faces in a subliminal affective priming task [18].

A key question in understanding vulnerability to depression is whether this abnormality in automatic negative processing is an enduring neurofunctional “trait” of depression or whether it is rather associated with the depressive state itself. Long-term neuroimaging studies have only examined changes in *controlled* stages of emotion processing, and suggest a normalization of activity in limbic regions in case of remission [19–21]. Regarding *automatic* emotion processing, there are no prospective neuroimaging studies examining the course of depression over periods longer than 8 weeks. Short-term studies indicate an initial normalization of amygdala dysfunction during subliminal emotion processing after psychopharmacological treatment and electroconvulsive therapy [17, 22–24], suggesting that also the early, automatic bias is a transient phenomenon that can be reversed by therapy. On the other hand, risk factors such as genetic susceptibility [25] or early maltreatment experiences [26] have both been associated with automatic processing biases in amygdala activation in healthy subjects, rather pointing to a trait-like vulnerability factor. Thus, it is unclear whether neurofunctional alterations during automatic emotion processing represent a stable correlate of depression in the long term.

Therefore, the main aim of our study was to investigate the neural correlates of automatic emotion processing of the course of depression over 2 years within a naturalistic study design. Due to its special role in early, automatic emotion processing and based on previous findings, a particular focus was placed on the amygdala. We used a well-established subliminal priming paradigm comprising emotional faces, which was designed to target emotion processing at its early (automatic) stage and to reliably evoke reactions in the amygdala [16, 26, 27]. The objectives of our study were as follows: (a) to replicate the mood-congruent bias on brain functional level during automatic emotion processing in patients with depression; (b) to examine changes in brain function during automatic emotion processing in dependence of the course of depression, more precisely, to investigate whether alterations in brain function normalize upon remission; and (c) to clarify if brain function during automatic emotion processing at baseline is predictive for the subsequent course of depression. Finally, we investigated the potential influence of treatments (medication intake and psychotherapy) during the study period on brain function.

## Method

### Participants and study design

Participants were examined from May 2010 to June 2015 (baseline) and reassessed after ~2 years from September 2012 to August 2017 (follow-up). The sample is part of an ongoing longitudinal study (Münster Neuroimaging Cohort).

At baseline, all patients suffered from an acute moderate or severe depressive episode and were under inpatient treatment at the Department of Psychiatry at the University Hospital of Münster or at the Psychiatric Hospital of the Landschaftsverband Westfalen-Lippe in Münster. The healthy control (HC) group was recruited through newspaper advertisements and public notices. Exclusion criteria were chronical medical illnesses, neurologic abnormalities, intake of benzodiazepine at any study time point, and any MRI contraindication. HC had to be free from any lifetime psychiatric disorder at both study points. MDD patients were excluded if diagnosed with bipolar or psychotic disorder, in case of acute substance dependence or with a history of electroconvulsive therapy. Diagnoses were verified at both study time points by trained clinical raters using the structured clinical interview for DSM-IV (SCID-I [28]). Details of the exclusion process are provided in Supplementary Fig. 1.

The final study sample comprised *n* = 57 patients with MDD and *n* = 37 HC. As described in our previous works [19, 29, 30], we divided the patient group into two subgroups, depending on their course if illness from baseline to follow-up (according to SCID-I criteria): Patients who were in ongoing depression at follow-up or experienced at least one depressive relapse between baseline and follow-up (*MDD relapse*-group; *n* = 37), and patients who were in full remission at follow-up without any further depressive episode after baseline (*MDD no-relapse*-group; *n* = 20). Demographic and clinical details of the sample are included in Table 1. Details on comorbidities can be found in Supplementary Table 1. There was an overlap of *n* = 87 participants with our previous study investigating long-term changes in brain function during *conscious* emotion processing, employing a different, unrelated fMRI paradigm [19].

**Table 1:** Demographic and clinical details of the sample.

Variable	MDD relapse *n* = 37	MDD no-relapse *n* = 20	Healthy controls *n* = 37		
	Mean (SD)	Mean (SD)	Mean (SD)	*p* value^a^	*p* value^b^
Interscan interval (years)	2.23 (0.30)	2.26 (0.22)	2.36 (0.30)	0.168	0.707
Demographics					
Age	38.43 (12.37)	32.95(10.67)	37.73 (8.99)	0.167	0.100
Sex (female/male), no. of patients	20/17	10/10	16/21	0.645^c^	0.770^c^
Symptom severity					
HDRS baseline	23.08 (4.87)	21.85 (3.42)	0.62 (1.04)	**<0.001**	0.321
HDRS follow-up	13.27 (7.92)	3.35 (4.03)	1.06 (2.00)	**<0.001**	**<0.001**
Clinical details at baseline					
Disease progression (first episode/recurrent), no. of patients	5/32	6/14	–	–	0.132^c^
Number of depressive episodes before baseline	5.05 (7.22)	3.45 (5.25)	–	–	0.385
Number of inpatient treatments before baseline	2.49 (1.91)	1.45 (0.95)	–	–	**0.008**
Duration of inpatient treatment before baseline (months)	3.33 (4.28)	1.60 (2.62)	–	–	0.064
Cumulative duration of depression before baseline (months)^a^	39.43 (41.22)	13.35 (14.59)	–	–	**0.001**
Acute comorbid disorder (yes/no), no. of patients	16/21	3/17	–	–	**0.031**^**c**^
Acute comorbid anxiety disorder (yes/no), no. of patients	12/25	3/17	–	–	0.154^c^
Clinical details at follow-up					
Disease progression (first episode/recurrent), no. of patients	2/35	6/14	–	–	**0.011**^**c**^
Number of depressive episodes between baseline and follow-up^d^	1.49 (0.87)	0.00 (0.00)	–	–	**<0.001**
Number of inpatient treatments between baseline and follow-up	0.54 (0.69)	0.00 (0.00)	–	–	**<0.001**
Remission status at follow-up (no remission/partial remission/full remission), no. of patients	15/13/9	0/0/20	–	–	**<0.001**^**c**^
Acute comorbid disorder (yes/no), no. of patients	16/21	3/17	–	–	**0.031**^**c**^
Acute comorbid anxiety disorder (yes/no), no. of patients	12/25	2/18	–	–	0.060^c^
Medical treatment					
No. of patients under psychopharmacological medication at baseline (yes/no)	35/2	19/1	–	–	0.948^c^
No. of patients under psychopharmacological medication at follow-up (yes/no)	27/10	8/12	–	–	**0.015**^**c**^
Medication load index at baseline	2.27 (1.24)	1.85 (0.81)	–	–	0.130
Medication load index at follow-up	1.59 (1.46)	0.60 (0.82)	–	–	**0.002**
Psychotherapeutic treatment					
Number of psychotherapeutic treatments before baseline (0/1/2/no information), no. of patients	10/11/3/13	7/4/0/9	–	–	0.319^c^
No. of patients under psychotherapeutic treatment during study interval^e^ (yes/no)	26/11	10/10	–	–	0.130^c^
Therapy method, no. of patients					
Cognitive behavioral therapy	12	7	–	–	–
Psychoanalytic psychotherapy	2	0	–	–	–
Psychodynamic psychotherapy	5	0	–	–	–
Other method	1	0	–	–	–
No information regarding method	17	13	–	–	–
No. of sessions during study interval	33.81 (30.42)	25.35 (33.25)	–	–	0.336

*HDRS* Hamilton Depression Rating Scale, *MDD* Major Depressive Disorder.

^a^Comparing patients with relapse, without relapse and healthy controls by using a one-way analysis of variance except where noted.

^b^Comparing patients with relapse and patients without relapse by using the unpaired two-tailed *t*-test except where noted.

^c^*p* values were obtained using the *Χ*²-test.

^d^Missing data for two subjects.

^e^Psychotherapeutic treatment during study interval was coded as yes with ≥12 sessions of psychotherapy.

Bold values indicate significant *p* values (p < .05).

At both study points, participants underwent fMRI while a subliminal affective priming paradigm was applied. Furthermore, SCID-I, [28] and Hamilton Depression Rating Scale (HDRS) [31] were conducted by trained clinical raters. Course of illness before and during study interval as well as type and dose of medication intake at both study points were documented. A composite medication load index (Supplementary Methods 1.1) was computed for each time point to quantify psychiatric medication intake at baseline and follow-up. Additionally, the frequency and type of psychotherapeutic interventions were documented (Table 1).

The study was approved by the ethics committee of the University of Münster (2007-307-f-S). All procedures comply with the ethical standards of the relevant national and institutional committees on human experimentation and with the Helsinki Declaration of 1975, as revised in 2008. All participants gave written informed consent and received financial compensation for study participation.

### Subliminal affective priming paradigm

In order to assess automatic emotion processing, a well-established subliminal affective priming paradigm [16, 18, 26] was used during fMRI. For detailed information on the paradigm and an illustration of an example trial, see Supplementary Methods 1.2 and Supplementary Fig. 2. Briefly, the paradigm consisted of 80 trials showing either a sad, happy, neutral face or no-face prime stimuli at subliminal perception level (duration: 33 ms), which was then masked by a neutral target face image of the same person at supraliminal perception level (duration: 467 ms). Face images were derived from the stimulus collection of Ekman and Friesen. Participants were asked to rate their impression of the emotional valence of each neutral target face from negative to positive on a four-point scale by pressing a button. Participants were unaware regarding the presence of emotional prime stimuli.

### FMRI methods

Acquisition and preprocessing of fMRI data followed previously published protocols [19, 32, 33].

### Data acquisition

T2* functional data were acquired using a single-shot echoplanar sequence with a 3T scanner (Gyroscan Intera 3T, Philips Medical System, Best, The Netherlands). Parameters were chosen to minimize distortion in the region of central interest retaining an adequate signal-to-noise ratio (S/N) and T2* sensitivity: 34 slices, matrix 64 × 64, resolution 3.6 × 3.6 × 3.6 mm; repetition time = 2.1 s, echo time = 30 ms, flip angle = 90°. Slices were acquired in an interleaved mode (first odd, then even), image numbering transversal F»H. To minimize dropout artifacts in the orbitofrontal and mediotemporal regions, slices were tilted 25° from the anterior and posterior commissure line. The paradigm was projected to the rear end of the scanner (Sharp XG-PC10XE with additional HF shielding; Osaka, Japan*)*, while participants lay supine in the MRI scanner.

### Data preprocessing

Functional data were realigned, unwarped and spatially normalized to the standard Montreal Neurological Institute (MNI) space. Images were smoothed with a Gaussian kernel of 6 mm full-width at half-maximum using Statistical Parametric Mapping software (SPM8; https://www.fil.ion.ucl.ac.uk/spm/).

Applying an event-related analysis design, trials were averaged separately for the happy, sad, neutral, and no-face prime condition for each participant and time point, reducing the data to four average trials per participant per time point. The onsets of the four prime conditions (positive, negative, neutral, no-face) were modeled by using a canonical haemodynamic response function. The model was corrected for serial correlations and a high-pass filter of 128 s was applied to remove low-frequency noise. Two individual first-level contrast images (sad > neutral, happy > neutral) were then used in the following second-level, random-effects group analyses. As we were interested in processing emotional stimuli, the no-face prime condition was not included in the analyses of this study.

### Statistical analyses

Clinical and demographic data were analyzed using SPSS Statistics (version 28.0.1.0 for Windows; IBM Corporation).

All second-level analyses of fMRI data were performed with Statistical Parametric Mapping software (SPM12, version 7771 for MATLAB on Ubuntu; Wellcome Department of Cognitive Neurology, London, UK; http://www.fil.ion.ucl.ac.uk/spm) and employed a region-of-interest (ROI) approach of the bilateral amygdala. The mask for the amygdala ROI was generated using the Wake Forest University PickAtlas20 toolbox (version 3.0; NeuroImaging Tools & Resources Collaboratory; https://www.nitrc.org/projects/wfu_pickatlas) within SPM12 according to the automated anatomical labeling atlas (version 3.1; [34]) definitions.

We used threshold-free cluster enhancement (TFCE) as a non-parametric approach for the ROI analyses in the amygdala, by using the TFCE toolbox (version 232; Structural Brain Mapping Group, Jena, Germany; http://dbm.neuro.uni-jena.de/tfce) implemented in SPM12. A combined peak-cluster-level family-wise error (FWE)-corrected threshold of *p* < 0.05 was applied to correct for multiple testing, obtained by 10,000 permutations per test.

We conducted a 3 × 2 × 2 analysis of covariance (ANCOVA) with group (HC, MDD relapse group, MDD no-relapse group) as between-subjects factor and time (baseline, follow-up) as well as condition (happy>neutral, sad>neutral) as within-subjects factors. Age and sex were included as covariates of no interest.

Main effects (group, condition, time) as well as interaction effects (group × condition, group × time, condition × time, group × condition × time) were investigated. To examine our study objectives, we further tested for baseline differences in amygdala activity by assessing the group × condition interaction effect at baseline, as we expected a mood-congruent bias in (acute) depression (*objective a*). Subsequently, *paired* post-hoc *t*-tests within each group were performed to investigate within-group differences between conditions (sad>neutral, happy>neutral) as well as *unpaired* post-hoc *t*-tests comparing groups for each condition. Moreover, the group × condition interaction at follow-up (including subsequent post-hoc *t*-tests) was conducted to test whether the bias in emotion processing was still present after 2 years (*objective b*). Finally, an *unpaired t*-test comparing amygdala activity of the two MDD groups at baseline was conducted for each prime condition separately to test the predictive value of baseline amygdala activity for subsequent relapse *(objective c*).

For exploratory reasons, all analyses were additionally performed at the whole-brain level, by establishing a FWE-corrected threshold of *p* < 0.05 at voxel level.

### Additional analyses

The Supplementary Methods provide a description of our additional analyses (a) controlling for acute comorbid (anxiety) disorders (Supplementary Methods 1.3.1), (b) investigating the effects of medication and psychotherapy on changes in brain function (Supplementary Methods 1.3.2), as well as examining effects of (c) the current mood state (Supplementary Methods 1.3.3), (d) prior disease progression (Supplementary Methods 1.3.4), and (d) environmental risk (Supplementary Methods 1.3.5) on amygdala activity. Details on analyses of behavioral data can be found in the Supplementary Methods 1.3.6.

## Results

### ROI analysis of amygdala

The 3 × 2 × 2 ANCOVA derived a significant main effect of group (left: *k* = 126, *F*_2_,_362_ = 6.23, *p*_TFCE-FWE_ < 0.001, *η*_p_² = 0.122; right: *k* = 198, *F*_2_,_362_ = 4.72, *p*_TFCE-FWE_ < 0.001, *η*_p_² = 0.089). Main effects of condition (*p*_TFCE-FWE_ > 0.999) and time (*p*_TFCE-FWE_ > 0.999) were not significant. A significant group × condition interaction emerged (right: *k* = 8, *F*_1_,_362_ = 11.40, *p*_TFCE-FWE_ = 0.040, *η*_p_² = 0.106), when both patient subgroups were considered together as one group. There was no significant group × time, condition × time or group × condition × time interaction effect on amygdala activity (all *p*_TFCE-FWE_ ≥ 0.456).

#### Replication of the automatic mood-congruent emotion processing bias (*objective a*)

**Fig. 1:**
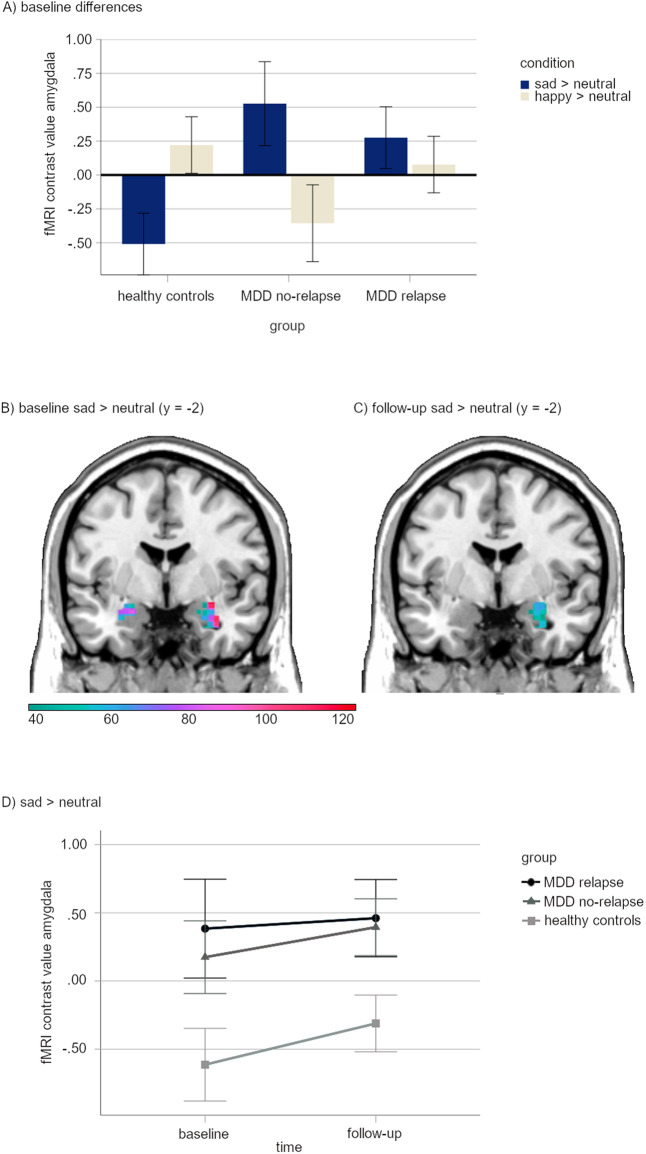
Results of the amygdala ROI analysis. **A** Bar graph depicting amygdala responses (sad > neutral) at baseline for the three groups. Error bars represent 1 SEM. fMRI contrast values were computed by extracting the first eigenvariate of the significant cluster resulting from the condition × group interaction effect (one-tailed) at baseline (*x* = −30, *y* = −6, *z* = −14, *t*_(362)_ = 3.40. *k* = 11, *p*_TFCE-FWE _= 0.027). **B** One-tailed *t*-test (HC > MDD) at baseline of the amygdala ROI analysis (right: *x* = 32, *y* = −2, *z* = −12, *t*_(362)_ = 4.08, *k* = 157 *p*_TFCE-FWE_ = 0.001; left: *x* = −24, *y* = 0, z = −16, *t*_(362)_ = 3.41, *k* = 110, *p*_TFCE-FWE_ = 0.005). Color bar indicates TFCE values. The figure displays clusters significant at *p*_TFCE-FWE_ < 0.05. Selected coronal brain slice (*y* = −2) represents the peak voxel. **C** One-tailed *t*-test (HC > MDD) at FU of the amygdala ROI analysis (right: *x* = 28, *y* = −6, *z* = −14, *t*_(362)_ = 2.94, *k* = 186 *p*_TFCE-FWE_ = 0.002; left: *x* = −28, *y* = −4, *z* = −22, *t*_(362)_ = 2.58, *k* = 8, *p*_TFCE-FWE_ = 0.036). Color bar indicates TFCE values. Figure displays clusters significant at *p*_TFCE-FWE_ < 0.05. For reasons of comparability, the selected coronal brain slice (*y* = −2) corresponds to the slice from Fig. 1B. Due to this, the left significant cluster (*k* = 8) is not visible in the figure. **D** Line graph illustrating changes in amygdala response for the three groups. Error bars indicate 1 SEM. fMRI contrast values were computed by extracting the first eigenvariate of the significant right cluster (*x* = 32, *y* = −2, *z* = −12, *t*_(362)_ = 4.08, *k* = 157, *p*_TFCE-FWE_ = 0.001) resulting from the amygdala ROI analysis of the one-tailed *t*-test (MDD > HC). MDD patients with major depressive disorder, ROI region of interest.

As stated in *objective a*, a significant group × condition interaction effect emerged in the bilateral amygdala at baseline (left: *k* = 17, *t*_*362*_ = 3.40, *p*_TFCE-FWE_ = 0.027; right: *k* = 14, *t*_*362*_ = 3.14, *p*_TFCE-FWE_ = 0.035). The post-hoc unpaired between-group *t*-tests revealed that both patient subgroups (MDD relapse and MDD no-relapse) showed significantly elevated amygdala activity to sad primes compared with HC (Table 2). For happy primes, there were no significant differences in amygdala activity between patients with depression and HC (all *p*_TFCE-FWE_ ≥ 0.644), albeit HC had nominally higher amygdala contrast values for happy face primes (Fig. 1).

**Table 2:** Cross-sectional group differences at baseline and FU for the amygdala region of interest.

Time point	Hemisphere	MNI-Coordinates (x,y,z)	*t*-value^a^	Cluster size *k*^b^	*p*_TFCE_-_FWE_ value
**Sad prime condition**					
**Baseline**	**MDD** > **HC**				
	right	32, −2, −12	4.08	157	**0.001**
	left	−24, 0, −16	3.41	110	**0.005**
	**No-relapse** > **HC**				
	right	32, −2, −12	3.80	6	**0.036**
	right	34, −2, −24	3.24	3	**0.047**
	**Relapse** > **HC**				
	right	30, 0, −14	3.53	85	**0.008**
	left	−22, 0, −16	3.36	71	**0.009**
	left	−22, −8, −12	2.38	1	**0.049**
**FU**	**MDD** > **HC**				
	right	28, −6, −14	2.94	186	**0.002**
	left	−28, −4, −22	2.58	8	**0.036**
	**No-relapse** > **HC**				
	right	32, 0, −26	2.68	18	**0.033**
	right	26, −8, −14	2.43	8	**0.046**
	**Relapse** > **HC**				
	right	24, 4, −16	2.99	119	**0.006**
	left	−30, −4, −22	2.64	21	**0.036**
	left	−24, −8, −16	2.39	1	**0.043**

*FWE* family-wise error corrected, *HC* healthy controls, *MDD* all patients with major depressive disorder, *MNI* coordinates of the peak-voxel of the significant cluster according to the standard Montreal Neurological Institute space, *TFCE* threshold-free cluster enhancement.

^a^Degrees of freedom for all *t*values were df = 362.

^b^Only significant clusters (*p*_TFCE_*-*_FWE_ < 0.05) are reported.

Bold values indicate significant *p* values (p < .05).

Paired *t*-tests within groups revealed that HC had higher amygdala activity to happy compared to sad primes (right: *k* = 45, *t*_*(362)*_ = 3.46, *p*_TFCE-FWE_ = 0.025). Patients with depression did not show significant differences in amygdala activity between happy and sad primes (all *p*_TFCE-FWE_ ≥ 0.214), with, however, higher contrast values for sad primes compared to happy primes in both patient subgroups (Fig. 1).

#### The automatic emotion processing bias in the long-term course of depression (*objective b*)

At follow-up, even though the group x condition interaction effect in the amygdala was not significant (*p*_TFCE-FWE_ = 0.107), both MDD subgroups continued to show elevated amygdala activity to sad primes compared with HC (Table 2). Surprisingly, for happy primes, the MDD relapse group showed even higher amygdala activity compared to HC at follow-up (left: *k* = 4, *t*_*(362)*_ = 2.66, *p*_TFCE-FWE_ = 0.046). The MDD relapse and the MDD no-relapse group did not differ significantly in amygdala activity in any condition at follow-up; however the relapse group tended to have higher amygdala activity to happy primes compared to the no-relapse group (*p*_TFCE-FWE_ = 0.058).

Paired *t*-tests within groups at follow-up revealed no differences in amygdala activity between happy and sad primes within any subgroup (all *p*_TFCE-FWE_ ≥ 0.222).

#### Baseline brain function and subsequent relapse (*objective c*)

The MDD relapse and the MDD no-relapse group did not differ in baseline amygdala activity neither in the sad prime (*p*_TFCE-FWE_ = 0.522), nor in the happy prime condition. (*p*_TFCE-FWE_ = 0.460).

### Additional analyses

#### Effect of comorbid (anxiety) disorders on amygdala activity

When additionally controlling for acute comorbid disorders in general and anxiety disorders in particular, the main effect of group and the group x condition interaction in the amygdala remained significant (Supplementary Results 2.1).

#### Effects of medication dose and psychotherapy on amygdala activity

There was a significant medication load × time interaction effect specifically for happy primes, indicating that higher medication loads were associated with elevated amygdala activity to happy primes only at follow-up, whereas at baseline, medication load and amygdala activity showed no significant associations (Supplementary Results 2.2). Our analyses showed no significant effect of psychotherapy on (changes in) amygdala function.

#### Effect of current mood state on amygdala activity

Briefly, there was no significant association between depressive symptom severity and amygdala activity (all *p* ≥ 0.296; Supplementary Results 2.3**)**. Additionally dividing patient groups by remission status at follow-up still revealed higher amygdala activity to sad primes in all patient subgroups compared to HC—independent of current remission status (Supplementary Table 2).

#### Effects of prior disease progression and environmental risk on amygdala activity

Patients with the first depressive episode at baseline did not differ in baseline amygdala activity to masked sad faces from patients in recurrence (*p*_TFCE-FWE_ = 0.190). There was a tendency for patients at high environmental risk (patients who had experienced childhood maltreatment) to have increased baseline amygdala activity to sad primes compared to patients without experience of childhood maltreatment (*p*_TFCE-FWE_ = 0.077).

#### Exploratory whole-brain analysis

At whole-brain level, a group × condition interaction effect emerged at baseline resulting from increased activity to sad primes in patients with MDD compared to HC in clusters including the middle temporal pole and middle temporal gyrus as well as the precentral, postcentral and angular gyrus and the superior occipital gyrus (Supplementary Table 3). At follow-up, groups did not differ in activity to sad primes on whole-brain level any more (*p*_FWE_ ≥ 0.362). When lowering the threshold to *p*_unc_ > 0.001, patient groups—particularly the relapse group—still showed increased activity to sad primes in salience and visual processing networks compared to HC (Supplementary Table 4). For details on exploratory whole-brain analyses, see Supplementary Results 2.4.

#### Behavioral results of affective priming task

At baseline, the MDD no-relapse group tended to rate neutral targets following sad primes more negatively compared to those followed by happy primes and showed a slight change towards a more positive rating of targets followed by sad primes at follow-up (Supplementary Results 2.5). However, the post-hoc *t*-test did not survive Bonferroni-correction. Reaction times in the sad prime condition compared to neutral prime condition were significantly longer at baseline, regardless of group.

## Discussion

The results of our study indicate that amygdala hyperactivity to masked negative stimuli persists in patients with depression after 2 years, regardless of relapse and current mood state. Thus, our results replicate the mood-congruent bias in depression during subliminal processing of emotional stimuli at the neural level, and furthermore suggest that this neural correlate of automatic emotion processing bias may be an enduring, trait-like feature of depression.

### Replication of the automatic mood-congruent emotion processing bias (*objective a*)

In line with prior cross-sectional studies investigating automatic emotion processing [15–17], patients with acute depression showed elevated amygdala activity during masked sad faces compared with HC, as well as nominally lower amygdala responsiveness to masked happy faces. Our results provide evidence of an altered pattern of automatic amygdala responses in depression during automatic stages of the emotional stimuli processing: While HC showed stronger responses to happy than to sad primes, amygdala activity of depressed patients did not differ between the two prime conditions, with however, higher contrast values for sad faces than for happy faces. Such a tendency to favor positive information during automatic processing in HC is consistent with the findings of previous studies [16, 35, 36], and points to a positive processing bias in individuals without mood disorders [15]. Our results indicate not only the absence of a positive processing bias in depressed patients, but even point to a negative bias in depression—thus a heightened sensitivity of the amygdala towards negative information even during early, automatic stages of emotion processing, as depressed patients showed heightened amygdala activity to masked sad faces compared with HC. These findings were independent of depression severity and comorbid (anxiety) disorders. In addition to its central role in fear conditioning [37], the amygdala is involved in reward-based learning [38]. Salience detection by the amygdala is processed by two parallel pathways: a conscious cortical circuit, as well as an unconscious subcortical circuit [39]. The dysfunctional patterns of automatic amygdala responses in depressed patients can lead to distortions of later, more conscious stages of information processing and negatively impact social behavior. This is in line with cognitive models of depression, stating that negative schemata influence information processing and lead to negative automatic thought patterns, which take impact on the individual’s feelings and behavior [2].

Interestingly, the same pattern of results was also found at the whole-brain level at baseline, pointing to an involvement of cortical regions in the negative automatic bias in emotion processing, such as the superior occipital gyrus, middle temporal gyrus, the temporal pole, the precentral and postcentral gyrus, and the angular gyrus. The occipital gyrus is part of the visual network and has shown a covariation with amygdala activity especially during condition with masked stimuli [39]. Furthermore, also the sensory cortex areas and temporal cortical areas have bidirectional connections to the amygdala and are as well involved in emotion processing [40]. The postcentral gyrus involves the somatosensory cortex which is a central hub for processing sensory information [41], and the angular gyrus plays an important role in attention and cognitive processes, particularly in integrating information from different sensory information [42]. These findings support the notion, that besides the amygdala, also further brain regions involved in early attention and visual perception processes underlie the negative bias in automatic emotion processing in patients with depression.

In contrast to psychological studies [18, 43], our analyses did not reveal affective priming effects at the behavioral level, as the prime condition had no effect on valence ratings. Instead, all participants showed longer reaction times to masked sad compared to masked neutral faces. As negative stimuli are evolutionarily more significant, sad primes may require more processing resources, even if presented subliminally. However, there is likewise contrary evidence of faster processing of negative stimuli [44].

Patients with depression also did not show significantly more negative valence ratings compared to HC. Even though there was a tendency for the MDD no-relapse group rating sad primes significantly more negative compared to happy primes at baseline and showing slightly more positive ratings at follow-up, this effect did not survive the Bonferroni correction and should therefore be interpreted with caution.

In summary, we could not clearly demonstrate the affective priming effect on behavioral level, although detectable on brain functional level. Possibly, functional neuroimaging may be more sensitive in measuring biases in subliminal emotion processing compared to behavioral assessments, supporting the relevance of brain functional imaging for investigating the dynamics of emotion processing.

### The automatic emotion processing bias in the long-term course of depression (*objective b*)

Our study reveals that the neurofunctional alterations during automatic emotion processing persist in the 2-year course of depression even in full remission: Both patient subgroups, independent of mood state and course of illness during study interval, still showed elevated amygdala activity to masked sad faces compared with HC. These findings stand in contrast to fMRI studies examining *controlled stages* of emotion processing employing supraliminal stimuli, that reported a normalization of brain functional alterations in case of remission [19–21]. Whereas brain functional alterations in later, controlled stages of emotion processing seem to be more sensitive and variable to the depressive state, the neurofunctional patterns of the early, automatic emotion processing bias seem to represent a stable “trait” marker of depression. Our findings are consistent with a previous cross-sectional study that found elevated amygdala activity to masked sad faces in patients in full remission compared to HC [17]. For happy primes, we could not find significantly reduced activity in patients with depression neither at baseline, nor at FU, which may imply that the bias in automatic emotion processing applies more strongly to negative primes.

However, it is unclear whether amygdala hyperactivity to negative stimuli develops as a consequence of or represents a risk marker for depression. Since all patients in our study were already depressed at baseline, increased amygdala activity to masked negative stimuli may be a result of the prior disease course and thus a scar effect of depression. For example, some brain structural studies showed no differences between subjects at risk of depression and HC or only found atrophy after the onset of depression [45, 46]. At least, our additional analyses revealed no significant difference between patients in their first depressive episode and patients in recurrence—however changes in amygdala activity could manifest as an early consequence of depression already in the first episode.

There are some indications that increased amygdala activity may represent a neural correlate of the vulnerability factors that underlie depression. For example, amygdala activity to automatic processing of subliminally presented negative stimuli has been found in association with childhood maltreatment [26], a relevant environmental risk factor for depression, as well as in carriers of the risk allele of the 5-HTTLPR polymorphism [25]. Furthermore, never-depressed daughters of depressed mothers already show attentional biases to negative facial expressions in dot-probe tasks [47, 48], supporting the notion that the negative bias in automatic emotion processing may be present in individuals at risk for developing depression already in early childhood. Consistent with this, we found that baseline amygdala activity to masked sad faces tended to be increased in patients who had experienced childhood maltreatment, suggesting a link between amygdala hyperactivity and risk factors for depression.

Whereas amygdala hyperactivity to negative stimuli persisted in both patient subgroups at follow-up, the difference in whole-brain activity between patients and HC was no longer significant after 2 years. In line with this, a cross-sectional study found that remitted patients with unipolar depression showed no difference in amygdala connectivity to other brain areas during subliminal emotion processing compared to HC—although amygdala activity itself was increased [49]. Possibly in our study, the conservative whole-brain FWE-corrected *p* < 0.05 significance threshold may have obscured potential differences between groups at follow-up. Lowering the threshold to *p*_unc_ < 0.001 for exploratory purposes suggested a still elevated activity to sad primes in regions of the salience and visual processing network in patients with depression, particularly the relapse group, compared to HC at follow-up. Together, these findings suggest that hyperactivity to subliminal negative processing represents a stable marker of depression, particularly in the amygdala. Abnormal functional patterns within other brain regions appear to be more closely tied to the depressive mood state, showing a degree of reversibility during periods of remission. Importantly, as the paradigm used in our study especially targets amygdala activity, the results at the whole-brain level should be interpreted carefully.

In contrast to our amygdala results, explicit treatment studies reported a normalization of the automatic emotion-processing bias in limbic regions after treatment with SSRIs [17, 22] and electroconvulsive therapy [24]. In line with this, another study found automatic amygdala activity to masked negative faces to be reduced after one week of citalopram intake in healthy individuals [50] indicating that antidepressants seem to act by reducing negative affective processing biases [51]. Even though we could not demonstrate these effects over the longer-term interval of 2 years in our study, our additional treatment analyses revealed that higher medication loads of psychiatric drugs were associated with elevated activity in the amygdala and caudate nucleus during the processing of masked happy faces. Higher activity in these regions during subliminal processing of happy primes may, therefore, reflect an elevated automatic sensitivity towards positive information and, therefore, a shift towards a positivity bias induced by medication.

These results may also explain why the MDD relapse group even showed higher amygdala activity towards happy primes compared to HC at follow-up. The MDD relapse group had significantly higher medication loads compared to the MDD no-relapse group at follow-up, so the differences may reflect a medication-induced effect rather than differences due to course of illness. Accordingly, a randomized placebo-controlled study showed that short-term treatment with citalopram increased amygdala activity to happy faces in healthy controls [52]. Alternatively, amygdala hyperactivity could also occur in a valence-unspecific manner during the progression of depression, so that the relapse group specifically shows increased amygdala activation at follow-up.

While HC showed significantly higher amygdala activity to happy compared to sad primes at baseline, we could no longer detect this positive bias at follow-up—possibly due to an adaptation to the priming paradigm in our study or due to a higher degree of variation in mood states at follow-up. Nominally, however, HC still showed higher activity to happy compared to sad primes at follow-up.

### Baseline brain function and subsequent relapse (*objective c*)

Contrary to our third hypothesis, brain activity at baseline did not differ between patient subgroups neither in the amygdala nor on whole-brain level. If replicated, this finding indicates that activity during automatic emotion processing does not appear to be a marker for predicting subsequent relapse. In contrast, in one of our earlier studies, we showed baseline differences in limbic activity between patients differing in their subsequent courses of illness [19]. These conflicting results may derive by different grades of consciousness during emotion processing, as in our earlier study, we investigated controlled stages of emotion processing using supraliminal stimuli. As automatic amygdala hyperactivity to negative stimuli seems to be a trait marker of depression in general, it may not be suitable for stratifying patient subgroups. Since our findings are preliminary and the sample size of the subgroup analyses is rather small, replication through additional long-term studies that specifically focus on automatic emotion processing is required.

#### Strengths and limitations

With a study interval of 2 years, this is the first prospective neuroimaging study examining changes in brain activity during automatic emotion processing in patients with depression over a period of more than 8 weeks. A well-established fMRI paradigm was implemented to measure the amygdala activity during the early, automatic stages of emotion processing. Also, the inclusion of a behavioral data analyses allows us a direct comparison of changes in the emotion processing bias over the depressive course over 2 years on brain functional and behavioral level. The naturalistic study design may allow a good transferability of the results to clinical reality.

Nevertheless, there are also some relevant limitations. The downside of a naturalistic study design is that there are factors that could have influenced the results, which can not be fully controlled (e.g., life events, prior course of illness, specific therapeutic interventions during study interval). It further has to be noted that our sample has an increased clinical severity, as all patients were hospitalized at baseline. About two-thirds relapsed or were still depressed after 2 years. Although there were no effects of first vs. recurrent episodes on baseline amygdala activity, it remains to be investigated whether our results also apply to patients with lower clinical severity. In addition, the sample size of patient subgroups is relatively small, potentially reducing the power of cross-sectional analyses in particular. Long-term fMRI studies with larger samples are challenging, but desirable.

In summary, our study indicates that automatic amygdala hyperactivity to subliminally presented negative stimuli persists even in the case of remission after 2 years. This marks a difference to later, more controlled stages of emotion processing and could be interpreted as an enduring trait marker for depression, possibly representing a neural correlate of vulnerability to or a consequence of depression.

### Supplementary information

Supplementary information

## Data Availability

The data that support the findings of this study are available from the corresponding author, RR, upon reasonable request.
