# Supplementary material for: Persistence of amygdala hyperactivity to subliminal negative emotion processing in the long-term course of depression
Source: Mol Psychiatry. 2024 Jan 26;29(5):1501–9. doi: 10.1038/s41380-024-02429-4 (PMC11189807; doi:10.1038/s41380-024-02429-4)
Supplement: Supplementary file 1 — Supplementary information [file 41380_2024_2429_MOESM1_ESM.docx]

**Supplementary information**

[1. Supplementary Methods 2](#_Toc153281925)

[1.1 Computation of the medication load index 2](#_Toc153281926)

[1.2 Affective priming paradigm 2](#_Toc153281927)

[1.3 Additional statistical analyses 2](#_Toc153281928)

[1.3.1 Effect of acute comorbid anxiety disorders on amygdala activity 2](#_Toc153281929)

[1.3.2 Effects of medication dose and psychotherapy 2](#_Toc153281930)

[1.3.3 Effect of current mood state on amygdala activity 3](#_Toc153281931)

[1.3.4 Effects of environmental risk for depression onset on amygdala activity 3](#_Toc153281932)

[1.3.5 Effects of disease progression before baseline on amygdala activity 3](#_Toc153281933)

[1.3.6 Behavioral data 4](#_Toc153281934)

[2. Supplementary Results 5](#_Toc153281935)

[2.1 Effects of comorbid anxiety disorders on amygdala activity 5](#_Toc153281936)

[2.2 Effects of medication dose and psychotherapy on amygdala activity 5](#_Toc153281937)

[2.3 Effect of current mood state on amygdala activity 5](#_Toc153281938)

[2.4 Results of the exploratory whole-brain analysis 5](#_Toc153281939)

[2.4.1 Baseline differences in whole-brain activity 6](#_Toc153281940)

[2.4.2 Changes in whole-brain activity from baseline to FU 6](#_Toc153281941)

[2.4.3 Differences in whole-brain activity at FU 6](#_Toc153281942)

[2.4.4 Effects of medication dose and psychotherapy on whole-brain activity 6](#_Toc153281943)

[2.5 Behavioral results of affective priming task 7](#_Toc153281944)

[2.5.1 Valence ratings 7](#_Toc153281945)

[2.5.2 Reaction time 7](#_Toc153281946)

[3. Supplementary Tables 9](#_Toc153281947)

[4. Supplementary Figures 14](#_Toc153281948)

[References 19](#_Toc153281949)

# 1. Supplementary Methods

## 1.1 Computation of the medication load index

The medication load index was computed according to the procedure described by Hassel et al. (1). Therefore, each psychotropic medication was coded as absent = 0, low = 1 (equal or lower average dose), or high = 2 (greater than average dose), relative to the midpoint of the daily dose range recommended by Physician’s-Desk-Reference (2). Then, all medication codes per participant and time point were summed, which finally yielded a composite measure of total medication exposure for each subject and time point (medication load index).

## 1.2 Affective priming paradigm

In each trial either a sad, happy, neutral face or no-face prime was presented at subliminal perception level (duration: 33ms), which was then masked by a neutral target face image of the same person at supraliminal perception level (duration: 467ms). Thereby, the neutral prime consisted of a vertical mirror-inversion of the neutral target face mask to avoid overlapping with the neutral target mask, whereas no-face primes consisted of stimuli with central facial features, such as the nose, mouth and eyes, replaced by a contourless surface. The paradigm comprised grey-scaled facial stimuli of 10 different persons (5 female, 5 male) originating from the stimulus collection of Ekman and Friesen (3) (resulting in 80 trials with 20 trials per condition (sad, happy, neutral, no-face).

The trials were presented in two versions in a pseudorandomized order (without showing the same person’s face or the same prime condition twice in succession). Each trial started with a fixation cross (800ms), followed by the prime (33ms) which was then replaced by the corresponding neutral target face mask (467ms) followed by a black screen (7.700ms). During the black screen, participants were asked to rate their impression of the emotional valence of the previously seen target face on a four-point scale by pressing a button. For this purpose, participants had a fiber-optic response pad with two buttons in each hand. For half of the participants, the negative pole was in the left and the positive pole in the right hand (-1.5, -.5, +.5, +1.5), for the other half it was reversed (+1.5, +.5, -1.5, -.5). Valence ratings as well as reaction times were registered. The total duration of the paradigm was 12 minutes, with each trial lasting 9 seconds.

## 1.3 Additional statistical analyses

### 1.3.1 Effect of acute comorbid (anxiety) disorders on amygdala activity

To rule out the possibility that the amygdala effects were due to comorbid psychiatric disorders in general and anxiety disorders in particular, we repeated our original group (HC, relapse, no-relapse) x time (baseline, follow-up) x condition (sad, happy) ANCOVA for the amygdala ROI in SPM: In two separate models, additionally to age and sex as covariates of no interest, we included acute comorbidity and acute comorbid anxiety disorders, each as a dummy-coded (0 = “no”; 1 = “yes”) covariate of no interest. Partially remitted comorbid (anxiety) disorders were also categorized as “yes”. In contrast, fully remitted comorbid (anxiety) disorders and the absence of a comorbid disorder were coded as "no". In our second analysis, we considered comorbid specific phobia, social phobia, agoraphobia, panic disorder, generalized anxiety disorder and posttraumatic stress disorder as anxiety disorders.

### 1.3.2 Effects of medication dose and psychotherapy

To account for potential treatment effects of medication and psychotherapy on brain function, we performed a subsequent group (relapse, no-relapse) x time (baseline, follow-up) analysis of covariance (ANCOVA), for each condition separately (happy >neutral, sad>neutral), including age and sex as covariates of no interest and including further covariates: a) the medication load index at baseline and follow-up, and b) psychotherapeutic treatment during study interval (dummy-coded: 1, “yes” ≥ 12 sessions, corresponding to a short-term therapy according to the German guidelines for psychotherapy [Psychotherapie-Richtlinie]; 2, “no” < 12 sessions). Main effects of treatments as well as treatment x time interaction effects were investigated. These analyses were conducted for the bilateral amygdala ROI and on whole-brain level.

### 1.3.3 Effect of current mood state on amygdala activity

In order to investigate whether increased amygdala activity to masked sad faces in patients with depression was associated with current depression severity, additional regression analyses were performed for the sad > neutral condition in SPM, with depression symptom severity (measured by the sum score of the Hamilton Depression Rating Scale; HDRS) as independent variable. Age and sex were included as covariates of no interest. These regression analyses were performed for the amygdala ROI across all depressed patients, and were conducted separately for baseline and follow-up.

To further explanatorily investigate the effects of remission status on follow-up amygdala activity, the relapse group was divided into two subgroups according to the current mood state at follow-up (relapse-acute vs. relapse-remitted). Then, a subsequent one-way ANOVA was computed in SPM for the amygdala ROI, including only the images of the two conditions (sad > neutral, happy > neutral) at follow-up as dependent variable and the factor subgroup as independent variable (HC, no-relapse, relapse-remitted, relapse-acute). The main effect of group and the group x condition effect were tested by *F*-tests, following by subsequent post-hoc *t*-tests comparing subgroups for each condition separately.

### 1.3.4 Effects of prior disease progression on baseline amygdala activity

To test whether increased amygdala activity to masked sad faces is be a function of prior disease progression, we conducted a one-tailed *t*-test in SPM for the sad > neutral condition at baseline comparing patients who were in the first depressive episode (*n* = 11) with patients in recurrence, i.e. with ≥ 2 lifetime depressive episodes (*n* = 46). Age and sex were included as covariates of no interest.

### 1.3.5 Effects of environmental risk for depression onset on baseline amygdala activity

To test whether increased amygdala activity is linked to an underlying risk marker of depression onset, we tested differences in amygdala activity at baseline between patients at low vs. high environmental risk of depression. Environmental risk for depression onset was operationalized by experience of childhood maltreatment, measured by the Childhood Trauma Questionnaire (CTQ; (4)) The CTQ is a commonly used self-report questionnaire to assess childhood maltreatment (5). It consists of five subscales: emotional abuse, emotional neglect, physical neglect, physical abuse and sexual abuse (4). The cutoff for each subscale was selected according to the definitions of Walker et al.(6). Patients were coded with a high environmental risk, when at least one of the CTQ subscale scores exceeded the respective cutoff-score, whereas patients with no CTQ subscale score over the respective cutoff-score were characterized as having a low environmental risk for depression onset. Then, a one-tailed *t*-test was conducted in SPM for the sad > neutral condition within the amygdala ROI in order to test whether patients with high environmental risk show elevated amygdala activity to sad primes compared to patients with low environmental risk. Age and sex were included as covariates of no interest.

### 1.3.6 Behavioral data

Behavioral response data of the affective priming paradigm were analysed by performing two group (HC, no-relapse, relapse) x time (baseline, follow-up) x condition (happy, sad, neutral) analyses of variance (ANOVA) in SPSS with response valence and reaction time as dependent variable, respectively.

Compound symmetry was verified by using Mauchly’s test. Variance homogeneity between groups was checked by Levene’s test for all ANOVAs.

Only subjects with available baseline and follow-up data were included. Due to technical issues, behavioral data of *n* = 17 subjects were missing at one or both time points as the fibre-optic cable did not transmit the response signal from the MR room to the control room in these cases. Missing values were imputed by the mean of response valence and reaction times within subject, respectively, with a maximum of 20% of missing data points per subject. In *n* = 7 subjects, more than 20% of the responses were missing due to technical issues. These subjects were excluded from the behavioral analyses (see **Supplementary Fig. 3**).

# 2. Supplementary Results

## 2.1 Effects of comorbid (anxiety) disorders on amygdala activity

When including acute comorbid disorder as a covariate into the 3x2x2 ANCOVA, the main effect of group (right: *k*=160, *F*_2_,_362_=5.39, *p*_TFCE-FWE_ = .003; left: *k*=98, *F*_2_,_361_=5.10, *p*_TFCE-FWE_ = .007) and the group x condition interaction (right: *k*=8, *F*_1_,_361_=11.38, *p*_TFCE-FWE_=.043) remained significant. There was no significant main effect of acute comorbid anxiety disorder (*p*_TFCE-FWE_ > .999).

Even when specifically controlling for anxiety disorders, there was still a significant main effect of group (left: *k*=157, *F*_2_,_362_=5.65, *p*_TFCE-FWE_ = .002; right: *k*=108, *F*_2_,_361_=5.24, *p*_TFCE-FWE_ = .003) and a significant group x condition interaction (right: *k*=6, *F*_1_,_361_=11.45, *p*_TFCE-FWE_=.045). There was no significant main effect of acute comorbid anxiety disorder (*p*_TFCE-FWE_ > .999).

## 2.2 Effects of medication dose and psychotherapy on amygdala activity

The majority of patients (*n* = 34; 60%) was under psychopharmacologic medication at both study time points, while around a third (*n* = 20; 35%) received medication at baseline but stopped medication intake during the study interval. A minor part either took no medication at any study time point (*n* = 2; 3%) or started medication during the study interval (*n* = 1; 2%). Two thirds of the patients (*n* = 36; 63%) were under psychotherapeutic treatment between baseline and follow-up with and were receiving an average of 31 sessions (*SD*=31.41).

For sad primes, neither medication load, nor psychotherapy during study interval was significantly associated with amygdala activity (all *p*_TFCE-FWE_’s > .999). Furthermore, there was no significant treatment x time interaction effect (all *p*_TFCE-FWE_’s > .999). For happy primes, there was a significant medication x time interaction effect (right: *k* = 8, *F_(_*_1, 104)_ = 10.85, *p*_TFCE-FWE_ = .042; *k* = 1, *F_(_*_1, 104)_ = 11.46, *p*_TFCE-FWE_ = .049) on amygdala activity. Post-hoc *t*-tests revealed a positive effect of medication load on amygdala activity only at FU (*k* = 4, *t_(_*_104)_ = 3.09, *p*_TFCE-FWE_ = .046), whereas at baseline, the association of medication with amygdala activity was not significant (*p*_TFCE-FWE_ =.081). There was no main effect of psychotherapy (*p*_TFCE-FWE_ > .999) or medication load (*p*_TFCE-FWE_ > .999), and no significant psychotherapy x time interaction effect (*p*_TFCE-FWE_ > .999).

## 2.3 Effect of current mood state on amygdala activity

The regression analyses revealed no significant association between HDRS score and amygdala activity to masked sad faces neither at baseline (*p*_TFCE-FWE_ = .202) nor at follow-up (*p*_TFCE-FWE_ = .358).

The one-way ANOVA with the four subgroups divided by relapse and remission status (HC, no-relapse, relapse-remitted, relapse-acute) revealed a significant main effect of group (left: *k*=43, *F*_3_,_178_=3.84, *p*_TFCE-FWE_ = .001; right: *k*=49, *F*_3_,_178_=4.61, *p*_TFCE-FWE_ < .001).

All patient subgroups (no-relapse, relapse-acute, relapse-remitted) showed amygdala hyperactivity to sad primes compared to HC (see **Supplementary Table 2**). Comparisons between depressive subgroups showed no significant differences for sad primes (all *p*_TFCE-FWE_ ≥ .362).

For happy primes, the relapse-acute group showed elevated amygdala activity compared to HC (see **Supplementary Table 2**) and compared to the no-relapse group (right: *k*=2, *t_(178)_*=3.95, *p*_TFCE-FWE_=.042). All other between-group comparisons for happy primes were not significant (*p*_TFCE-FWE_ > .187)

## 2.4 Results of the exploratory whole-brain analysis

The 3x2x2 ANCOVA revealed no significant main effect of group (*p*_FWE_ = .335), condition (*p*_FWE_ > .999) or time (*p*_FWE_ > .999). Two-way interactions of group x condition (*p*_FWE_ = .099), group x time (*p*_FWE_ = .752) and condition x time (*p*_FWE_ = .870) and the three-way group x condition x time interaction were not significant (*p*_FWE_ = .977).

### 2.4.1 Baseline differences in whole-brain activity

At baseline, a directional group x condition interaction effect emerged at baseline in clusters including the middle temporal pole and middle temporal gyrus as well as the precentral, postcentral and angular gyrus and the superior occipital gyrus (for details see **Supplementary Table 1**): More specifically, patients with major depressive disorder (MDD) had increased activity to sad primes compared with healthy controls (HC) in the left middle temporal gyrus (*k* = 3, *t_(362)_* = 4.77, *p* = .042) and in the right postcentral gyrus (*k* = 2, *t_(362)_* = 4.74, *p*_FWE_ = .047), while there was no difference between groups in whole-brain activity to happy primes (all *p*_FWE_ ≥ .713). Whole brain activity did not differ between the MDD relapse and the MDD no-relapse group in any condition (all *p*_FWE_ ≥ .279).

### 2.4.2 Changes in whole-brain activity from baseline to FU

There was no significant main effect of time (*p*_FWE_ *>* .999) and no significant group x time or group x time x condition interaction effect at the whole-brain level (all *p*_FWE_ ≥ .752).

### 2.4.3 Differences in whole-brain activity at FU

At FU, there was no significant group x condition interaction effect at the whole-brain level (*p*_FWE_ *>* .999). Furthermore, groups did not differ in whole-brain activity at FU – neither in the sad prime condition (all *p*_FWE_ ≥ .362) nor in the happy prime condition (all *p*_FWE_ ≥ .143).

In order to detect potential between-group differences in whole-brain activity to sad primes that did not survive the conservative FWE-corrected threshold of *p*<.05, we exploratory lowered our significance threshold at whole-brain level to *p*_unc_ < .001. These exploratory analyses revealed elevated activity to sad faces in the relapse group compared to HC also at follow-up in several regions including e.g. the fusiform and lingual gyrus, cingulate gyrus, frontal cortex, insula, thalamus, cuneus and precuneus (for results, see **Supplementary Table 4**). Also the no-relapse group still showed elevated activity to sad primes compared to healthy controls. There were furthermore differences in brain activity between the two patient groups at follow-up, pointing to elevated activity to sad primes in the relapse group compared to the no-relapse group in the gyrus rectus and the middle frontal gyrus.

### 2.4.4 Effects of medication dose and psychotherapy on whole-brain activity

For sad primes, neither medication load, nor psychotherapy during study interval were significantly associated with whole-brain activity (all *p*_FWE_’s ≥ .399). There was no significant treatment x time interaction effect (all *p*_FWE_’s ≥ .170). For happy primes, a main effect of medication load emerged in the right nucleus caudatus (*k* = 6, *F*_(1,140)_ = 31.31, *p*_FWE_ = .012). The post-hoc *t*-tests revealed a positive association between medication load and right nucleus caudatus activity at baseline (*k* = 9, *t_(104_)* = 5.60, *p*_FWE_ = .006). At FU, there was no significant effect of medication on whole-brain activity (*p*_FWE_ > .999). There was no main effect of psychotherapy (*p*_FWE_ = .337) nor significant psychotherapy x time interaction effect (*p*_FWE_ ≥ .198).

## 2.5 Behavioral results of affective priming task

For the behavioral analyses, *n* = 70 subjects were included (*n* = 24 HC, *n* = 19 no-relapse, *n* = 27 relapse).

### 2.5.1 Valence ratings

Group means and standard deviations of valence ratings for each time point and condition are indicated in **Supplementary Table 5.** The repeated measures ANOVA with condition (sad, happy, neutral) and time (baseline, follow-up) as within-subjects factors and group (no-relapse, relapse, HC) as between-subjects factor revealed the following results: Compound symmetry (all *p*’s ≥ .467) and homogeneity of variance were given (all *p*’s ≥ .189).

The repeated measures ANOVA revealed a significant condition x time x group interaction (*F*_(4,134)_ = 2.528, *p* = .044, partial *η*² = .070). Pairwise post-hoc *t*-tests within groups revealed that only the no-relapse group showed a shift to a better valence of sad stimuli specifically (*t*_(18)_ = 2.529, *p*_unc_ = .021, Cohen’s *d* = 0.580, see **Supplementary Table 6**). However, applying a Bonferroni-corrected threshold *p* = 0.0056 (9 post-hoc tests), this effect was no longer significant. There was no significant main effect (all *p*’s ≥ .330) and no other significant interaction effect (all *p*’s ≥ .204).

When computing a repeated-measures group x condition ANOVA for both time points separately, the following results emerged: Compound symmetry (all *p*’s ≥ .060) and variance homogeneity were given (all *p*’s ≥ .189) in both repeated-measures ANOVAs. At baseline, a significant group x condition effect emerged (*F*_(4,134)_ = 2.480, *p* = .047, partial *η*² = .069, see **Supplementary Fig. 4**). Post-hoc *t*-tests within groups revealed that the no-relapse group rated stimuli with sad primes significantly more negative compared to those with happy primes (*t*_(18)_= 2.871, *p*_unc_ = .010, Cohen’s *d* = 0.659), whereas there were no significant differences in valence ratings between conditions in the healthy control group (all *p*_unc_’s ≥ .113) or in the MDD relapse group (all *p*_unc_’s ≥ .252). However, this difference did not survive the Bonferroni-corrected threshold of *p* = .0083 (6 post-hoc tests).

The repeated-measures ANOVA at baseline revealed no main effect of condition (*F*_(2, 134)_ = 1.944, *p* = .147) and no main effect of group (*F*_(2, 67)_ = 0.398, *p* = .673). At follow-up, there was no significant main effect or interaction effect (all *p*’s ≥ .423, see **Supplementary Fig. 5**).

### 2.5.2 Reaction time

Group means and standard deviations of valence ratings for each time point and condition are indicated in **Supplementary Table 7**. The Mauchly’s test for sphericity was significant for the time x condition interaction (Mauchly’s *W*_(2)_ = 0.826, *p*=.002). In this case, the Huynh-Feldt (HF)-correction was applied. For the other effects, compound symmetry (*p* = .413) or variance homogeneity (all *p*’s ≥ .569) were given. Our analyses yielded a significant main effect of condition (*F*_(2, 134)_ = 11.990, *p* <.001, partial *n*² = .152). Pairwise comparisons independent of groups revealed that at baseline, participants had longer reaction times to sad primes compared to happy primes and compared to neutral primes (see **Supplementary Table 8**). These differences in reaction times were still present at follow-up. Reaction times were also significantly longer to happy primes compared to neutral primes, however only at baseline. However, applying a Bonferroni-corrected threshold of *p* = .0083 (6 post-hoc tests), only the effect of longer reaction times to sad compared to neutral primes at baseline remained significant.

There was a significant time x condition interaction effect (*F*_(1.796, 120.317)_ = 3.262, *p* = .047, partial *n*² = .137). Pairwise *t*-tests for each condition independent of group revealed that reaction times to sad primes were significantly shorter at follow-up compared to baseline (*t*_(69)_=2.393, *p*_unc_ = .019, Cohen’s *d* = 0.286), whereas there was no change in reaction times for the two other conditions (all *p*’s ≥ .062). This effect did not survive the Bonferroni-corrected threshold of *p* = .0167 (3 post-hoc tests). Our analyses did not show any further significant main effect or interaction effect (all *p*’s ≥ .075). Bar graphs for the reaction times separated by group and condition are depicted for baseline in **Supplementary Fig. 6** and for follow-up reaction times in **Supplementary Fig. 7**.

# 3. Supplementary Tables

**Supplementary Table 1 Acute psychiatric comorbidities in the patient groups at both study time points**

| **Acute psychiatric comorbidities,**  **no. of patients^1^** | **MDD relapse**  ***n*=37** | **MDD no-relapse**  ***n*=20** |
| --- | --- | --- |
| Baseline |  |  |
| Anxiety disorders |  |  |
| Agoraphobia | 3 | 0 |
| Panic disorder | 4 | 2 |
| Social phobia | 5 | 0 |
| Specific phobia | 2 | 0 |
| Generalized anxiety disorder | 3 | 1 |
| Posttraumatic stress disorder | 2 | 0 |
| Others |  |  |
| Dysthymia | 4 | 0 |
| Obsessive compulsive disorder | 2 | 0 |
| Eating disorder | 2 | 0 |
| Substance abuse | 0 | 0 |
| Follow-up |  |  |
| Anxiety disorders |  |  |
| Agoraphobia | 4 | 1 |
| Panic disorder | 3 | 1 |
| Social phobia | 5 | 1 |
| Specific phobia | 3 | 0 |
| Generalized anxiety disorder | 0 | 0 |
| Posttraumatic stress disorder | 2 | 0 |
| Others |  |  |
| Dysthymia | 4 | 0 |
| Obsessive compulsive disorder | 0 | 0 |
| Eating disorder | 3 | 0 |
| Substance abuse | 1 | 1 |

*Abbreviations*: MDD = major depressive disorder.
^1^Multiple comorbidities per patient possible.

**Supplementary Table 2 Cross-sectional group differences in amygdala activity at FU, with groups subdivided according course of depression and remission status at FU**

| **Condition** | **Hemisphere** | | **MNI-Coordinates (x,y,z)** | ***t*-value^1^** | **Cluster size *k*^2^** | ***p*_TFCE_-_FWE_ value** |
| --- | --- | --- | --- | --- | --- | --- |
| **Sad primes** | **No-relapse > HC^3^** | |  |  |  |  |
|  | Right | | 32 0 -26 | 2.86 | 19 | **.036** |
|  | Right | | 26 -8 -14 | 2.64 | 8 | **.044** |
|  | **Relapse-acute > HC^3^** | | |  |  |  |
|  | Right | | 24 2 -12 | 3.31 | 96 | **.011** |
|  | Left | | -30 -4 -22 | 3.01 | 6 | **.038** |
|  | Left | | -24 2 -16 | 2.66 | 2 | **.049** |
|  | **Relapse-remitted > HC^3^** | |  |  |  |  |
|  | Right | | 24 4 -16 | 2.75 | 13 | **.038** |
| **Happy primes** | **No-relapse > HC^3^** | | – | – | – | .276 |
|  | **Relapse-acute > HC^3^** | |  |  |  |  |
|  | Left | -28 -2 -16 | | 3.12 |  | **.022** |
|  | **Relapse-remitted > HC^3^** | | – | – | – | ..187 |

*Abbreviations*: FWE = Family-wise error corrected, HC = healthy controls, MNI = Coordinates of the peak-voxel of the significant cluster according to the standard Montreal Neurological Institute space, TFCE = Threshold-free cluster enhancement.

^1^Degrees of freedom for all *t*-values were df=178.

^2^Only significant clusters (*p*_TFCE_*-*_FWE_<.05) are reported.

**Supplementary Table 3 Results of the group x condition interaction at baseline at whole-brain level**

| **Anatomical region** | **Hemi-sphere** | **MNI-Coordinates (x,y,z)** | **t-value^1^** | **Cluster size *k*** | ***p*_FWE_-value** |
| --- | --- | --- | --- | --- | --- |
| Superior occipital gyrus | Right | 24, -90, 30 | 5.22 | 4 | **.006** |
| Middle temporal pole / middle temporal gyrus | Right | 54, 8, -26 | 5.16 | 4 | **.008** |
| Precentral gyrus | Left | -34, 2, 44 | 5.04 | 4 | **.014** |
| Angular gyrus | Right | 46, -58, 34 | 4.94 | 4 | **.021** |
| Postcentral gyrus | Left | -36, -24, 42 | 4.94 | 3 | **.021** |
| Cerebellum | Right | 26, -64, -26 | 4.78 | 1 | **.040** |
| Cuneus | Right | 10, -74, 36 | 4.75 | 1 | **.045** |

*Abbreviations*: FWE = Family-wise error corrected, MNI = Coordinates according to the standard Montreal Neurological Institute space.

^1^ Degrees of freedom of all *t*-values were df=362.

**Supplementary Table 4 Whole-brain results of the independent *t*-tests (exploratory analyses with *p*_unc_ < .001) between subgroups for sad primes at follow-up**

| **Post-hoc *t*-test** | **Anatomical region** | **Hemi-sphere** | **MNI-Coordinates (x,y,z)** | **t-value^1^** | **Cluster size *k*** | ***p*_unc_-value** |
| --- | --- | --- | --- | --- | --- | --- |
| **Relapse > HC** | Cerebellum / Fusiform gyrus / Lingual gyrus | Right | 12, -58, -30 | 4.15 | 155 | **<.001** |
|  | Fusiform gyrus / Cerebellum | Right | 40, -50, -22 | 4.07 | 82 | **<.001** |
|  | Cingulate gyrus, posterior part / Thalamus / Precuneus | Right / Left | -2, -36, 10 | 3.95 | 68 | **<.001** |
|  | Superior temporal gyrus / Middle temporal gyrus | Right | 48, -18, -8 | 3.94 | 58 | **<.001** |
|  | Precentral gyrus / Middle frontal gyrus | Right | 48, 6, 38 | 3.88 | 38 | **<.001** |
|  | Cerebellum / Vermis | Right | 10, -74, -20 | 3.71 | 31 | **<.001** |
|  | Cingulate gyrus, posterior part / Precuneus | Right | -2, -50, 24 | 3.69 | 51 | **<.001** |
|  | Inferior frontal gyrus, orbital / Inferior frontal gyrus, triangular / Insula | Right | 36, 34, -2 | 3.69 | 35 | **<.001** |
|  | Superior temporal gyrus / Heschl gyrus / Middle temporal gyrus / Insula | Left | -46, -30, 6 | 3.65 | 104 | **<.001** |
|  | Calcarine fissure and surrounding cortex / Precuneus / Cuneus / Lingual gyrus | Right / Left | 2, -66, 16 | 3.64 | 84 | **<.001** |
|  | Insula | Left | -32, 16, 0 | 3.63 | 76 | **<.001** |
|  | Parahippocampal gyrus / Lingual gyrus / Fusiform gyrus | Left | -14, -36, -6 | 3.58 | 34 | **<.001** |
| **No-relapse > HC** | Cingulate gyrus, posterior part | Right / Left | 0, -40, 18 | 3.59 | 30 | **<.001** |
| **Relapse > no-relapse** | Gyrus rectus / Middle frontal gyrus, orbital part | Left | -8, 46, -16 | 3.29 | 8 | **.001** |

*Abbreviations*: unc = uncorrected for multiple comparisons, MNI = Coordinates according to the standard Montreal Neurological Institute space.

^1^ Degrees of freedom of all *t*-values were df=362.

^2^ For reasons of comprehensibility, only clusters with k>30 are reported.

**Supplementary Table 5 Behavioral results of priming task: Group means (and standard deviations) of valence ratings (scale: -1.5, -0.5, 0.5, 1.5)**

|  | **Baseline** | | |  | **Follow-up** | | |
| --- | --- | --- | --- | --- | --- | --- | --- |
| **Condition** | **MDD relapse**  ***n* = 27** | **MDD no-relapse**  ***n* = 19** | **HC**  ***n* = 24** |  | **MDD relapse**  ***n* = 27** | **MDD  no-relapse**  ***n* = 19** | **HC**  ***n* = 24** |
| Sad | -0.080 (0.255) | -0.167 (0.205) | -0.062 (0.245) |  | -0.073 (0.400) | 0.009 (0.191) | -0.056 (0.323) |
| Happy | -0.057 (0.267) | -0.074 (0.244) | -0.082 (0.241) |  | -0.067 (0.404) | -0.012 (0.195) | -0.059 (0.324) |
| Neutral | -0.089 (0.265) | -0.126 (0.120) | -0.044 (0.210) |  | -0.046 (0.411) | -0.041 (0.205) | -0.073 (0.374) |

*Abbreviations*: MDD = Major depressive disorder, HC = healthy controls.

**Supplementary Table 6 Behavioral results of priming task: paired *t*-tests of valence ratings within groups**

|  | **Sad** |  | **Happy** |  | **Neutral** |  |
| --- | --- | --- | --- | --- | --- | --- |
| **Contrast** | ***t*-value** | ***p*-value^1^** | ***t*-value** | ***p*-value^1^** | ***t*-value** | ***p*-value^1^** |
| HC: pre vs. FU | 0.108 | .915 | 0.373 | .712 | 0.486 | .632 |
| No-relapse: pre vs. FU | **2.529*** | **.021*** | 0.956 | .352 | 1.360 | .190 |
| Relapse: pre vs. FU | 0.82 | .935 | 0.111 | .913 | 0.463 | .647 |

*Abbreviations*: FU = follow-up, HC = healthy controls.

^1^ *p*-values are two-tailed, uncorrected for multiple comparisons. Degrees of freedom for all *t*-tests were df = 26.

**Supplementary Table 7 Behavioral results of priming task: Group means (and standard deviations) of reaction times (in ms)**

|  | **Baseline** | | |  | **Follow-up** | | |
| --- | --- | --- | --- | --- | --- | --- | --- |
| **Condition** | **MDD relapse**  ***n* = 27** | **MDD no-relapse**  ***n* = 19** | **HC**  ***n* = 24** |  | **MDD relapse**  ***n* = 27** | **MDD  no-relapse**  ***n* = 19** | **HC**  ***n* = 24** |
| Sad | 1618.722 (333.164) | 1567.525 (344.031) | 1520.659 (383.396) |  | 1553.076 (393.750) | 1497.943 (368.236) | 1423.382 (328.537) |
| Happy | 1550.463 (384.265) | 1538.387 (385.225) | 1487.845 (449.251) |  | 1462.794 (301.098) | 1459.168 (356.211) | 1422.601 (316.539) |
| Neutral | 1520.296 (328.745) | 1481.760 (363.548) | 1423.258 (342.432) |  | 1490.180 (344.435) | 1464.211 (379.712) | 1408.440 (317.642) |

*Abbreviations*: MDD = Major depressive disorder, HC = healthy controls.

**Supplementary Table 8 Behavioral results of priming task: paired t-tests of reaction times between conditions over all groups**

|  | **Baseline** |  | **Follow-up** |  |
| --- | --- | --- | --- | --- |
| **Contrast** | ***t*-value** | ***p*-value^1^** | ***t*-value** | ***p*-value^1^** |
| Sad vs. happy | 2.377* | **.020** | 2.298* | **.025** |
| Sad vs. neutral | 5.354*** | **<.001** | 2.588* | **.012** |
| Happy vs. neutral | 2.172* | **.033** | 0.472 | .638 |

^1^ *p*-values are two-tailed, uncorrected for multiple comparisons. Degrees of freedom for all *t*-tests were df = 69.

# 4. Supplementary Figures

**Supplementary Fig. 1. Flowchart illustrating the exclusion process from the Muenster Neuroimaging Cohort resulting in the final study sample**

Subjects with fMRI paradigm
and SCID-I
at baseline and after two years

*n* = 53 HC, *n* = 83 MDD

Subjects with complete data
at baseline and follow-up
meeting inclusion criteria

*n =* 44 HC*, n =* 72 MDD

**Final study sample**

Excluded because of

- ECT between baseline
and follow-up (*n* = 10 MDD)

- HC with depressive episode between baseline and
follow up (*n* = 8 HC)

- neuroanatomical abnormalities
(*n* = 1 HC, *n* = 1 MDD)

Excluded because of
excessive head movement
(> 3mm / 3°)

*n =* 7 HC*, n =* 15 MDD

HC

*n* = 37

MDD no-relapse

*n* = 37

MDD

*n* = 57

MDD relapse

*n* = 20

*Abbreviations*: ECT = electroconvulsive therapy; fMRI = functional magnetic resonance imaging; HC = healthy controls; MDD = major depressive disorder; MDD no-relapse = patients with major depressive disorder in full remission at follow-up and no further depressive episode after baseline; MDD relapse = patients with major depressive disorder in acute depression at follow-up or further episode after baseline; SCID-I = structured clinical interview for DSM-IV.

**Supplementary Fig. 2. Example trial of fMRI paradigm**


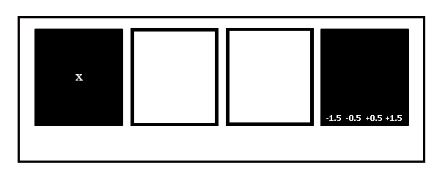


*Prime:
33 ms*

*Mask:
467 ms*

*Black screen:
7 700 ms*

*Fixation cross:
800 ms*

Happy, sad, neutral face expression or no-face prime

Neutral face expression of the same person

**Supplementary Fig. 3. Flowchart illustrating the exclusion process for the behavioral data analyses**

Study sample with complete
fMRI data

*n* = 37 HC

*n* = 37 MDD relapse

*n* = 20 MDD no-relapse

Sample with available behavioral data

*n* = 26 HC

*n* = 32 MDD relapse

*n* = 19 MDD no-relapse

Sample for behavioral analyses

*n* = 24 HC

*n* = 27 MDD relapse

*n* = 19 MDD no-relapse

Missing behavioral data due to technical issues

*n* = 11 HC

*n* = 5 MDD relapse

*n* = 1 MDD no-relapse

Exclusion from behavioral analyses due to > 20% missing values, due to technical issues

*n* = 2 HC

*n* = 5 MDD relapse

*n* = 0 MDD no-relapse

*Abbreviations*: fMRI = functional magnetic resonance imaging; HC = healthy controls; MDD = major depressive disorder; MDD no-relapse = patients with major depressive disorder in full remission at follow-up and no further depressive episode after baseline; MDD relapse = patients with major depressive disorder in acute depression at follow-up or further episode after baseline.

**Supplementary Fig. 4. Bar diagram of behavioral responses (valence ratings) at baseline**

Estimated marginal means of participants, for each group and condition. Error bars indicate 1 s.e.m. Valence ratings were given by participants on a 4-point-scale from -1.5 (very negative) to +1.5 (very positive).


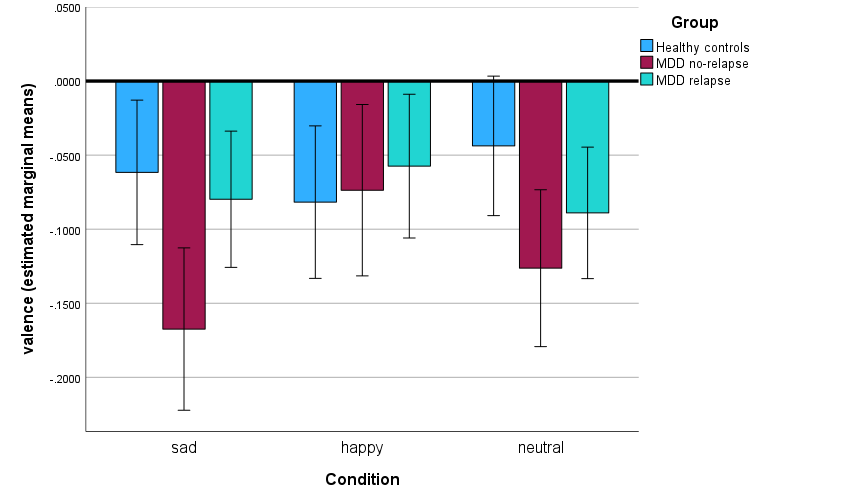


**Supplementary Fig. 5. Bar diagram of behavioral responses (valence ratings) at follow-up**

Estimated marginal means of participants, for each group and condition. Error bars indicate 1 s.e.m. Valence ratings were given by participants on a 4-point-scale from -1.5 (very negative) to +1.5 (very positive).


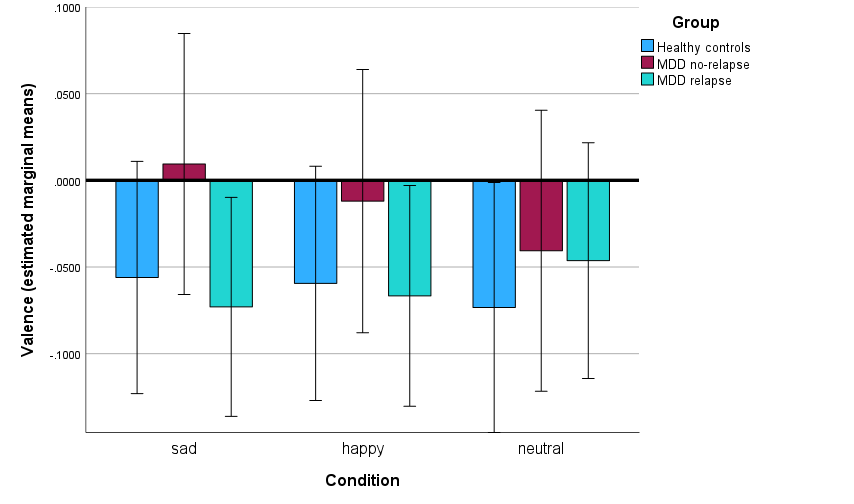


**Supplementary Fig. 6. Bar diagram of reaction times of behavioral responses at baseline**

Estimated marginal means of reaction times in ms, for each group and condition. Error bars indicate 1 s.e.m.

**
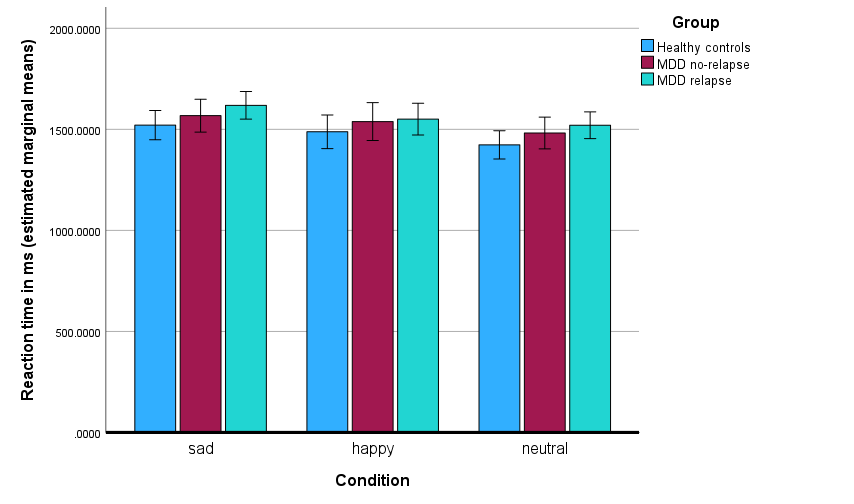
**

**Supplementary Fig. 7. Bar diagram of reaction times of behavioral responses at follow-up.**

Estimated marginal means of reaction times in ms, for each group and condition. Error bars indicate 1 s.e.m.


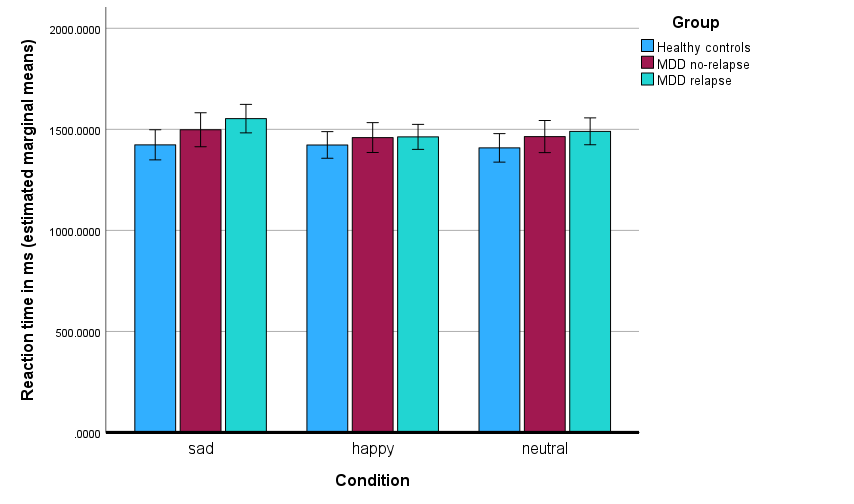


# References

1. Hassel S, Almeida JRC, Kerr N, Nau S, Ladouceur CD, Fissell K, et al. Elevated striatal and decreased dorsolateral prefrontal cortical activity in response to emotional stimuli in euthymic bipolar disorder: no associations with psychotropic medication load. Bipolar Disorders. 2008;10(8):916–27.

2. Reynolds CR. Physician’s Desk Reference. In: Reynolds CR, Fletcher-Janzen E, editors. Encyclopedia of Special Education. Vol Hoboken, NJ, USA: John Wiley & Sons, Inc.; 2008.

3. Ekman P, Friesen WV. Pictures of Facial Affect. Palo Alto, CA: Consulting Psychologists Press; 1976.

4. Bernstein D, Fink L, Handelsman L, Foote J, Lovejoy M, Wenzel K, et al. Initial reliability and validity of a new retrospective measure of child abuse and neglect. Am J Psychiatry. 1994 Aug;151(8):1132-6. Am J Psychiatry. 1994 Aug;151(8):1132–6.

5. Viola TW, Salum GA, Kluwe-Schiavon B, Sanvicente-Vieira B, Levandowski ML, Grassi-Oliveira R. The influence of geographical and economic factors in estimates of childhood abuse and neglect using the Childhood Trauma Questionnaire: A worldwide meta-regression analysis. Child Abuse & Neglect. 2016 Jan;51:1–11.

6. Walker EA, Gelfand A, Katon WJ, Koss MP, Von Korff M, Bernstein D, et al. Adult health status of women with histories of childhood abuse and neglect. The American Journal of Medicine. 1999 Oct;107(4):332–9.
